# Health-care expenditures are less for minimally invasive than open colectomy for colon cancer: A US commercial claims database analysis

**DOI:** 10.1007/s00464-023-10104-y

**Published:** 2023-05-16

**Authors:** Amir L. Bastawrous, I.-Fan Shih, Yanli Li, Marissa Khalil, Biruk Almaz, Robert K. Cleary

**Affiliations:** 1 Swedish Cancer Institute, Swedish Health System, Seattle, WA USA; 2 Global Health Economics and Outcomes Research, Intuitive Surgical, Inc, Sunnyvale, CA USA; 3 Vanderbilt University School of Medicine, Nashville, TN USA; 4 Department of Surgery, St Joseph Mercy Hospital Ann Arbor, 5325 Elliott Dr. Suite 104, Ann Arbor, MI 48106 USA

**Keywords:** Colon resection, Colon cancer, Cost, Health-care utilization, Minimally invasive, Robotic-assisted surgery

## Abstract

**Background:**

Most studies comparing surgical platforms focus on short-term outcomes. In this study, we compare the expanding societal penetration of minimally invasive surgery (MIS) with open colectomy by assessing payer and patient expenditures up to one year for patients undergoing surgery for colon cancer.

**Methods:**

We analyzed the IBM MarketScan Database for patients who underwent left or right colectomy for colon cancer between 2013 and 2020. Outcomes included perioperative complications and total health-care expenditures up to 1 year following colectomy. We compared results for patients who had open colectomy (OS) to those with MIS operations. Subgroup analyses were performed for adjuvant chemotherapy (AC+) versus no adjuvant chemotherapy (AC-) groups and for laparoscopic (LS) versus robotic (RS) approaches.

**Results:**

Of 7,063 patients, 4,417 cases did not receive adjuvant chemotherapy (OS: 20.1%, LS: 67.1%, RS: 12.7%) and 2646 cases had adjuvant chemotherapy (OS: 28.4%, LS: 58.7%, RS: 12.9%) after discharge. MIS colectomy was associated with lower mean expenditure at index surgery and post-discharge periods for AC- patients (index surgery: $34,588 vs $36,975; 365-day post-discharge $20,051 vs $24,309) and for AC+ patients (index surgery: $37,884 vs $42,160; 365-day post-discharge $103,341vs $135,113; *p* < 0.001 for all comparisons). LS had similar index surgery expenditures but significantly higher expenditures at post-discharge 30 days (AC-: $2,834 vs $2276, *p* = 0.005; AC+: $9100 vs $7698, *p* = 0.020) than RS. The overall complication rate was significantly lower in the MIS group than the open group for AC- patients (20.5% vs 31.2%) and AC+ patients (22.6% vs 39.1%, both *p* < 0.001).

**Conclusion:**

MIS colectomy is associated with better value at lower expenditure than open colectomy for colon cancer at the index operation and up to one year after surgery. RS expenditure is less than LS in the first 30 postoperative days regardless of chemotherapy status and may extend to 1 year for AC- patients.

**Supplementary Information:**

The online version contains supplementary material available at 10.1007/s00464-023-10104-y.

Colon cancer is a common indication for colectomy. Based on concerns for port site recurrences, a moratorium in the late 1990s and early 2000s limited the adoption of the laparoscopic approach for colorectal cancer. The 2004 COST trial showed non-inferiority of laparoscopy compared to open surgery for colon cancer and demonstrated the safety of the laparoscopic approach [1]. Other studies confirmed this finding and a new era of minimally invasive (MIS) colectomy was ushered in [2, 3]. However, a decade passed before the MIS approach became more common than the open approach for diseases of the colon [4–6].

While numerous studies have shown the safety and efficacy of MIS in colon cancer, most have focused on short-term outcomes advantages that include shorter hospital length of stay (LOS), fewer surgical site infections (SSI), less postoperative ileus, and lower opioid use [6–9]. Health-care environment financial pressures have led some to question the value of minimally approaches due to higher intraoperative costs. We previously showed that surgical health-care expenditures were significantly less for the MIS than the open approach for benign colon diseases [10]. Malignant diagnoses were excluded in that study to limit the confounding effect of expenses such as systemic chemotherapy or variable presentations that were unique to colon cancer.

The aim of this study was to assess total value of elective MIS versus open (OS) and laparoscopic (LS) versus robotic (RS) surgical approaches for colon cancer by examining short- and long-term financial impact in the form of health-care expenditures. We also compared the impact of adjuvant chemotherapy on surgical approach choice.

## Materials and methods

### Data source

This is a retrospective cohort study using data from the IBM® MarketScan® Commercial Claims and Encounter Database (MarketScan®), an aggregated database that captures paid claims and encounter data generated by approximately 50 million US commercially insured individuals. This database contains the enrollment and medical claims of inpatient, outpatient, and prescription drug services from employees and their dependents insured by employer-sponsored plans [11]. As this was an observational study of de-identified claims in the MarketScan®, Institutional Review Board approval and consent were exempt based on the Health Insurance Portability and Accountability Act Privacy Rule.

### Study population

All adults aged 18 to 64 years diagnosed with colon cancer and underwent an inpatient colectomy between January 2013 and December 2019 were identified from MarketScan®. *International Classification of Diseases and Related Health Problems, Ninth* and *Tenth Revisions, Clinical Modification and Procedure Classification System (ICD-9-CM/ICD-10-CM/ ICD-9-PCS/ICD-10-PCS*) were used to define the eligible colectomy cases and differentiate surgical approaches (Supplementary Table 1). To ensure complete follow-up, only patients who had continuously enrolled with medical and prescription drug coverage from 180 days prior to and 365 days after inpatient colectomy were included for data analysis. We further excluded: (1) emergent cases; (2) patients with neoadjuvant chemotherapy; (3) metastatic tumor diagnosis; (4) total colectomy; (5) inpatient cases that were not coded with diagnosis-related group (DRG) codes 329, 330, or 331; or (6) discharges with extreme total payment during index hospitalization (< 1st or > 99th). We defined emergent cases as patients who had an emergency room service claim found on the day of admission and neoadjuvant chemotherapy as any chemotherapy claims during the baseline period (180 days prior to inpatient colectomy). Patients were divided into 2 groups (AC+ vs. AC-) based on whether they had adjuvant chemotherapy during the follow-up period (365 days after discharge) based on ICD chemotherapy administration codes.

### Outcomes

We evaluated all inpatient, outpatient, and drug claims during the inpatient colectomy stay (index surgery) and up to 365-day post-discharge. The health-care expenditures included both facility and professional allowed payments by summing payer reimbursement and patient out-of-pocket payments. All expenditures were inflation adjusted to 2020 US dollars using the general Consumer Price Index. Complications during index surgery and within 30 days after discharge were identified based on ICD codes in any inpatient or outpatient service claims. These complications include overall complication rate, anastomotic leak, ileus, bleeding, and infection. ICD codes used to identify these complications and conversion-to-open rates are shown in Supplemental Table 2.

### Patient factors

Patient-level baseline sociodemographic and clinical characteristics included age, gender, region, metropolitan/non-metropolitan area, insurance plan, surgical site (Left: left hemicolectomy and sigmoidectomy; Right: right hemicolectomy and cecectomy), other concomitant indications (benign neoplasm, diverticular disease, inflammatory bowel disease), DRG codes, Charlson Comorbidity Index (CCI; without cancer score), obesity/overweight, year of surgery, and baseline total payment 180 days prior to index surgery. Insurance plans were classified into comprehensive insurance, preferred provider organization (PPO), capitated plans, non-capitated point of service (POS), and other insurance plans. DRG codes are listed in Table 1 but not included in statistical model for outcomes comparison. Obesity/overweight status and CCI were assessed during the index hospitalization and in the 180-day preoperative period.

**Table 1:** Baseline demographic characteristics before inverse probability of treatment weighting (IPTW) adjustment

Patients without adjuvant chemotherapy													
	Open (N = 888)	MIS (N = 3529)	SMD	LS (N = 2966)	RS (N = 563)	SMD		Open (N = 751)	MIS (N = 1895)	SMD	LS (N = 1554)	RS (N = 341)	SMD
	N (%)	N (%)		N (%)	N (%)			N (%)	N (%)		N (%)	N (%)	
Age, years			0.21			0.06				0.09			0.08
18–44	75 ( 8.4)	294 ( 8.3)		241 ( 8.1)	53 ( 9.4)			110 (14.6)	276 (14.6)		218 (14.0)	58 (17.0)	
45–54	254 (28.6)	1344 (38.1)		1122 (37.8)	222 (39.4)			259 (34.5)	732 (38.6)		605 (38.9)	127 (37.2)	
55–64	559 (63.0)	1891 (53.6)		1603 (54.0)	288 (51.2)			382 (50.9)	887 (46.8)		731 (47.0)	156 (45.7)	
Gender, Male	436 (49.1)	1749 (49.6)	0.01	1458 (49.2)	291 (51.7)	0.05		370 (49.3)	1007 (53.1)	0.08	820 (52.8)	187 (54.8)	0.04
Region			0.13			0.08				0.15			0.19
North Central	214 (24.1)	765 (21.7)		643 (21.7)	122 (21.7)			184 (24.5)	406 (21.4)		330 (21.2)	76 (22.3)	
Northeast	126 (14.2)	574 (16.3)		485 (16.4)	89 (15.8)			92 (12.3)	297 (15.7)		231 (14.9)	66 (19.4)	
South	446 (50.2)	1663 (47.1)		1396 (47.1)	267 (47.4)			386 (51.4)	916 (48.3)		757 (48.7)	159 (46.6)	
West	99 (11.1)	507 (14.4)		423 (14.3)	84 (14.9)			82 (10.9)	264 (13.9)		224 (14.4)	40 (11.7)	
Unknown	3 ( 0.3)	20 ( 0.6)		19 ( 0.6)	1 ( 0.2)			7 ( 0.9)	12 ( 0.6)		12 ( 0.8)	0 ( 0.0)	
Metropolitan			0.27			0.15				0.18			0.09
Yes	658 (74.1)	2918 (82.7)		2435 (82.1)	483 (85.8)			563 (75.0)	1551 (81.8)		1263 (81.3)	288 (84.5)	
No	199 (22.4)	438 (12.4)		390 (13.1)	48 ( 8.5)			158 (21.0)	268 (14.1)		225 (14.5)	43 (12.6)	
Unknown	31 ( 3.5)	173 ( 4.9)		141 ( 4.8)	32 ( 5.7)			30 ( 4.0)	76 ( 4.0)		66 ( 4.2)	10 ( 2.9)	
Insurance Plan			0.09			0.16				0.13			0.14
PPO	478 (53.8)	1940 (55.0)		1654 (55.8)	286 (50.8)			386 (51.4)	1058 (55.8)		865 (55.7)	193 (56.6)	
Capitated plan	126 (14.2)	427 (12.1)		338 (11.4)	89 (15.8)			97 (12.9)	235 (12.4)		189 (12.2)	46 (13.5)	
Comprehensive	38 ( 4.3)	172 ( 4.9)		143 ( 4.8)	29 ( 5.2)			44 ( 5.9)	71 ( 3.7)		55 ( 3.5)	16 ( 4.7)	
Non-cap POS	78 ( 8.8)	281 ( 8.0)		237 ( 8.0)	44 ( 7.8)			66 ( 8.8)	153 ( 8.1)		134 ( 8.6)	19 ( 5.6)	
Others	157 (17.7)	643 (18.2)		534 (18.0)	109 (19.4)			143 (19.0)	351 (18.5)		288 (18.5)	63 (18.5)	
Unknown	11 ( 1.2)	66 ( 1.9)		60 ( 2.0)	6 ( 1.1)			15 ( 2.0)	27 ( 1.4)		23 ( 1.5)	4 ( 1.2)	
Surgical site			0.12			0.19				0.08			0.18
Right	502 (56.5)	2207 (62.5)		1898 (64.0)	309 (54.9)			352 (46.9)	968 (51.1)		819 (52.7)	149 (43.7)	
Left	386 (43.5)	1322 (37.5)		1068 (36.0)	2547 (45.1)			399 (53.1)	927 (48.9)		735(47.3)	192 (56.3)	
IBD	24 ( 2.7)	75 ( 2.1)	0.04	61 ( 2.1)	14 ( 2.5)	0.03		16 ( 2.1)	24 ( 1.3)	0.07	21 ( 1.4)	3 ( 0.9)	0.05
Polyps	359 (40.4)	1540 (43.6)	0.07	1308 (44.1)	232 (41.2)	0.06		174 (23.2)	437 (23.1)	0.00	361 (23.2)	76 (22.3)	0.02
Diverticular	73 ( 8.2)	255 ( 7.2)	0.04	213 ( 7.2)	42 ( 7.5)	0.01		49 ( 6.5)	111 ( 5.9)	0.03	90 ( 5.8)	21 ( 6.2)	0.02
DRG			0.23			0.05				0.21			0.07
329	96 (10.8)	172 ( 4.9)		139 ( 4.7)	33 ( 5.9)			84 (11.2)	123 ( 6.5)		104 ( 6.7)	19 ( 5.6)	
330	623 (70.2)	2539 (71.9)		2140 (72.2)	399 (70.9)			619 (82.4)	1572 (83.0)		1282 (82.5)	290 (85.0)	
331	169 (19.0)	818 (23.2)		687 (23.2)	131 (23.3)			48 ( 6.4)	200 (10.6)		168 (10.8)	32 ( 9.4)	
Charlson Comorbidity			0.12			0.02				0.10			0.07
0	491 (55.3)	1993 (56.5)		1679 (56.6)	314 (55.8)			436 (58.1)	1048 (55.3)		866 (55.7)	182 (53.4)	
1	228 (25.7)	1018 (28.8)		855 (28.8)	163 (29.0)			200 (26.6)	590 (31.1)		484 (31.1)	106 (31.1)	
> = 2	169 (19.0)	518 (14.7)		432 (14.6)	86 (15.3)			115 (15.3)	257 (13.6)		204 (13.1)	53 (15.5)	
Overweight/Obesity	180 (20.3)	689 (19.5)	0.02	561 (18.9)	128 (22.7)	0.09		129 (17.2)	317 (16.7)	0.01	251 (16.2)	66 (19.4)	0.08
Year			0.19			0.71				0.30			0.54
2013	143 (16.1)	448 (12.7)		426 (14.4)	22 ( 3.9)			126 (16.8)	248 (13.1)		232 (14.9)	16 ( 4.7)	
2014	171 (19.3)	665 (18.8)		620 (20.9)	45 ( 8.0)			164 (21.8)	327 (17.3)		293 (18.9)	34 (10.0)	
2015	187 (21.1)	645 (18.3)		568 (19.2)	77 (13.7)			129 (17.2)	305 (16.1)		261 (16.8)	44 (12.9)	
2016	139 (15.7)	514 (14.6)		429 (14.5)	85 (15.1)			135 (18.0)	271 (14.3)		216 (13.9)	55 (16.1)	
2017	105 (11.8)	478 (13.5)		371 (12.5)	107 (19.0)			84 (11.2)	259 (13.7)		198 (12.7)	61 (17.9)	
2018	76 ( 8.6)	389 (11.0)		286 ( 9.6)	103 (18.3)			63 ( 8.4)	249 (13.1)		183 (11.8)	66 (19.4)	
2019	67 ( 7.5)	390 (11.1)		266 ( 9.0)	124 (22.0)			50 ( 6.7)	236 (12.5)		171 (11.0)	65 (19.1)	
Baseline total payment	$15,690 ± $19,859	$14,590 ± $19,157	0.06	$14,663 ± $19,568	$14,206 ± $16,835	0.03		$16,834 ± $23,499	$15,335 ± $17,279	0.07	$15,003 ± $17,142	$16,848 ± $17,837	0.11

IPTW, inverse probability of treatment weighting; SMD, standard mean difference; MIS, minimally invasive surgery; LS, laparoscopic surgery; RS, robotic surgery; PPO, preferred provider organization; Non-cap POS, Non-capitated Point of Service; DRG, Diagnosis-Related Group; DRG 329/330/331, major small and large bowel procedures. DRG codes were listed but not included in IPTW model

### Statistical analysis

All descriptive and statistical testing analyses were conducted comparing the open surgical approach to MIS approaches and subgroup analysis between LS and RS. Patient characteristics at baseline were summarized as frequencies with proportions for categorical variables and means with standard deviations for continuous variables. To minimize the effect of potential confounding factors without reducing the sample size, we performed inverse probability of treatment weighting (IPTW) with stabilized weights [12]. Propensity score weights were calculated from logistic regression models with all the baseline patient factors mentioned above except for DRG as covariates based on prior knowledge and literature [10, 13, 14]. After IPTW, covariates were considered balanced if the absolute value of the standardized mean difference (SMD) was less than 0.1. Generalized linear model and logistic regression weighted by the IPTWs and adjusting for the total health-care expenditures in the 180-day preoperative period (i.e., baseline expenditures) were used to compare the health-care expenditures and complication rates. All analyses were performed using SAS software version 9.4 (SAS Institute Inc., Cary, NC) and a 2-tailed *P* < 0.05 was considered statistically significant.

## Results

**Fig. 1:**
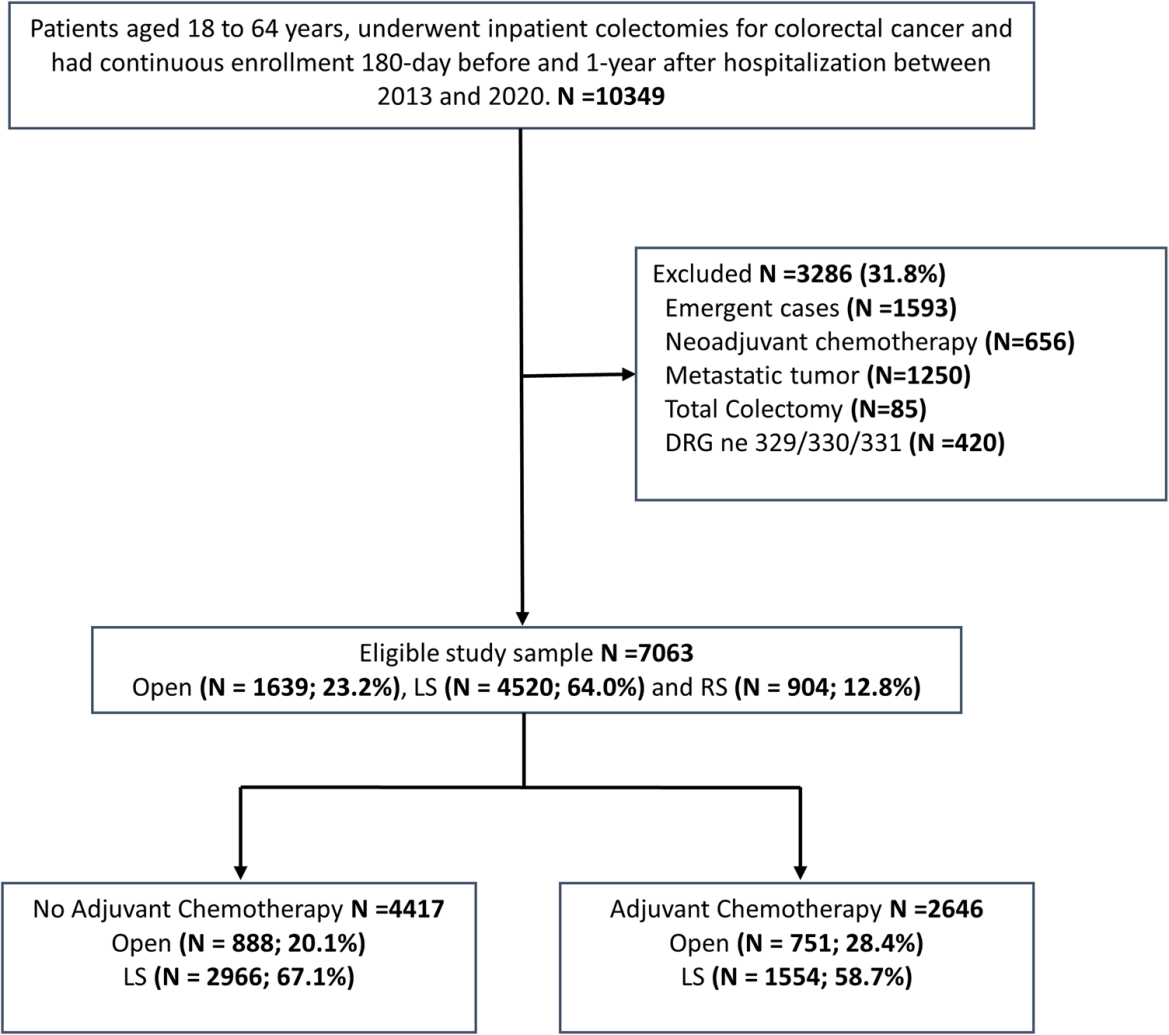
Study Flow. DRG, Diagnosis-Related Group; LS, laparoscopic surgery; RS, robotic surgery

A total of 7,063 elective inpatient colectomies for colon cancer were analyzed. Of these, 4417 cases did not receive adjuvant chemotherapy [AC-, 888 (20.1%) OS, 2966 (67.1%) LS, and 563 (12.7%) RS] and 2646 cases had adjuvant chemotherapy [AC +, 751 (28.4%) OS, 1554 (58.7%) LS, and 341 (12.9%) RS] after discharge (Fig. 1). Table 1 shows baseline sociodemographic characteristics before IPTW. Several characteristics were observed unbalanced before IPTW, including age, region, metropolitan, insurance plan, surgical site, DRG, CCI, and year of surgery. After IPTW, there was slight imbalance in the Region variable in patients with adjuvant chemotherapy (|SMD|= 0.13). All other sociodemographic variables were balanced between open and MIS and between LS and RS groups (Supplementary Table 3).

**Fig. 2:**
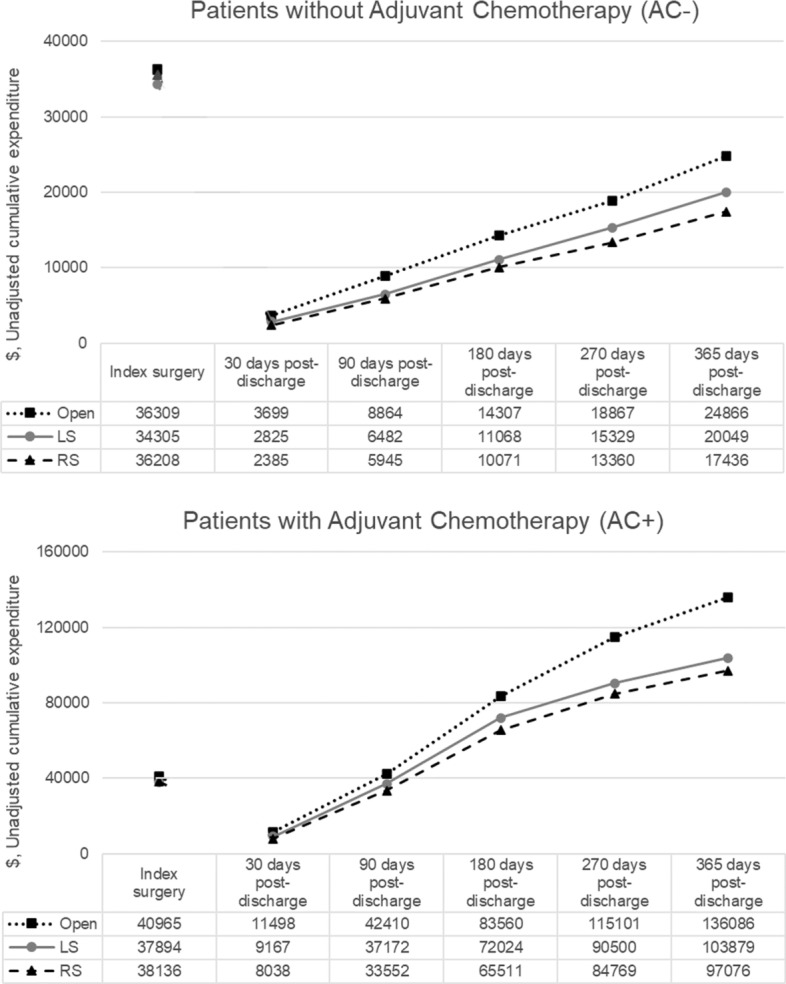
Time series graphics for the unadjusted expenditures. Health-care expenditure was calculated by adding hospital and physician payments during the inpatient stay (index surgery) and all health service-related costs within the 1 year after discharge, including inpatient, outpatient, and prescription drug services cumulatively. LS, laparoscopic surgery; RS, robotic surgery

Unadjusted mean health-care expenditures at index surgery hospital stay and cumulative expenditures from discharge to 365-day post-discharge by surgical modality are plotted in Fig. 2. In general, mean total expenditures were higher for AC+ patients than AC- patients at all time periods analyzed. When compared across surgical approaches, open patients had higher mean expenditures than LS and RS patients in both AC- and AC+ groups.

In IPTW-adjusted analyses, the mean health-care expenditures were significantly higher for open colectomy than for MIS colectomy at index surgery as well as at all post-discharge periods (Table 2): among AC- patients, index surgery episode ($36,975 vs $ 34,588), from discharge to 30-day post-discharge ($3661 vs $2748), 90-day post-discharge ($8492 vs $6451), 180-day post-discharge ($13,840 vs $11,056), 270-day post-discharge ($18,398 vs $15,256), and 365-day post-discharge ($24,309 vs $20,051); among patients with AC+, index surgery episode ($42,160 vs $ 37,884), from discharge to 30-day post-discharge ($11,423 vs $9024), 90-day post-discharge ($41,909 vs $36,797), 180-day post-discharge ($83,086 vs $71,318), 270-day post-discharge ($114,199 vs $90,050), and 365-day post-discharge ($135,113 vs $103,341, p < 0.001 for all comparisons). The difference in mean total health-care expenditures including index surgery and 1-year follow-up between open colectomy vs MIS was $6677 for AC- group and $36,048 for AC + group (both *p* < 0.001).

**Table 2:** IPTW-adjusted differences in health-care expenditures

	Open		MIS		Difference (Open—MIS)		LS		RS		Difference (LS—RS)	
	Mean	95%CI	Mean	95%CI	Mean	P-Value	Mean	95%CI	Mean	95%CI	Mean	P-Value
Without Adjuvant Chemotherapy												
Index surgery	36,975	(35,915, 38,066)	34,588	(34,088, 35,095)	2387	< .001	34,470	(33,937, 35,011)	35,427	(34,178, 36,722)	-957	0.174
30-day post-discharge	3661	(3244, 4130)	2748	(2587, 2919)	912	< .001	2834	(2654, 3027)	2276	(1956, 2648)	558	0.005
90-day post-discharge	8492	(7664, 9410)	6451	(6128, 6791)	2041	< .001	6480	(6127, 6853)	5810	(5107, 6609)	670	0.114
180-day post-discharge	13,840	(12,611, 15,188)	11,056	(10,553, 11,583)	2784	< .001	11,075	(10,531, 11,648)	9694	(8632, 10,886)	1382	0.031
270-day post-discharge	18,398	(16,860, 20,077)	15,256	(14,603, 15,937)	3143	< .001	15,291	(14,585, 16,032)	13,045	(11,699, 14,545)	2247	0.006
365-day post-discharge	24,309	(22,471, 26,298)	20,051	(19,276, 20,856)	4259	< .001	20,096	(19,262, 20,967)	17,043	(15,453, 18,797)	3053	0.001
Index + 1 year	61,215	(58,956, 63,560)	54,539	(53,522, 55,575)	6677	< .001	54,481	(53,406, 55,577)	52,301	(49,956, 54,755)	2180	0.105
With Adjuvant Chemotherapy												
Index surgery	42,160	(40,838, 43,524)	37,884	(37,131, 38,652)	4276	< .001	38,022	(37,208, 38,854)	37,982	(36,256, 39,791)	39	0.968
30-day post-discharge	11,423	(10,436, 12,505)	9024	(8524, 9553)	2400	< .001	9100	(8547, 9688)	7698	(6729, 8807)	1401	0.020
90-day post-discharge	41,909	(39,798, 44,131)	36,797	(35,619, 38,015)	5112	< .001	36,814	(35,544, 38,130)	34,074	(31,597, 36,744)	2740	0.062
180-day post-discharge	83,086	(79,053, 87,324)	71,318	(69,117, 73,589)	11,768	< .001	71,209	(68,831, 73,669)	67,591	(62,833, 72,709)	3618	0.197
270-day post-discharge	114,199	(108,661, 120,019)	90,050	(87,273, 92,915)	24,149	< .001	89,594	(86,681, 92,605)	87,417	(81,423, 93,852)	2177	0.535
365-day post-discharge	135,113	(128,512, 142,053)	103,341	(100,131, 106,654)	31,772	< .001	103,023	(99,666, 106,493)	99,731	(92,878, 107,091)	3291	0.413
Index + 1-year	177,273	(170,490, 184,324)	141,225	(137,796, 144,739)	36,048	< .001	141,045	(137,518, 144,662)	137,714	(130,420, 145,415)	3331	0.432

IPTW, inverse probability of treatment weighting; MIS, minimally invasive surgery; LS, laparoscopic surgery; RS, robotic surgery; CI, confidence interval

Subgroup analysis of LS vs RS shows comparable health-care expenditures at index surgery for AC- patients ($34,470 vs $ 35,427, *p* = 0.174) and AC+ patients ($38,022 vs $ 37,982, *p* = 0.968), while higher expenditures at post-discharge 30 days were observed for the LS approach (AC-: $2834 vs $2276, *p* = 0.005; AC+: $9100 vs $7698, *p* = 0.020) than for RS. In addition, within AC- patients, the mean expenditures were higher for the LS approach than RS from discharge to 180-day post-discharge (mean difference, $1382, *p* = 0.031), 270-day post-discharge (mean difference, $2247, *p* = 0.006), and 365-day post-discharge (mean difference, $3053, *p* = 0.001). There were no significant differences between LS and RS among AC + patients for expenditures beyond post-discharge 30-day timeframes (Table 2).

The overall rate of complication was significantly lower in the MIS group than in the open group for AC- patients (20.5% vs 31.2%) and AC + patients (22.6% vs 39.1%, both *p* < 0.001). Similar trends of lower rates of anastomotic leak, ileus, bleeding, and infection among MIS patients were also observed (*p* < 0.05 for all comparisons). Subgroup analysis of LS vs RS shows comparable complication rates except that RS has a lower overall complication rate among AC- patients that approaches but does not reach statistical significance (LS vs RS: 20.6% vs 17.0%, OR, 1.26 [1.00–1.60], *p* = 0.054). Conversion-to-open rates were significantly higher for the LS than for the RS group for AC- patients (5.5% vs 2.0%, *p* < 0.001) and AC + patients (8.9% vs 5.5%, *p* = 0.042) (Table 3).

**Table 3:** IPTW-adjusted postoperative complications

	Open	MIS	OR	95%CI	P-Value	LS	RS	OR	95%CI	P-Value
*Without Adjuvant Chemotherapy*										
Overall Complication	31.2%	20.5%	1.76	(1.50, 2.08)	< .001	20.6%	17.0%	1.26	(1.00, 1.60)	0.054
Anastomotic leak	9.2%	6.4%	1.48	(1.13, 1.93)	0.004	6.5%	5.3%	1.25	(0.84, 1.87)	0.267
Ileus	14.2%	8.3%	1.84	(1.47, 2.30)	< .001	8.6%	6.3%	1.41	(0.98, 2.03)	0.067
Bleeding	9.7%	7.3%	1.38	(1.07, 1.78)	0.014	7.1%	6.6%	1.08	(0.75, 1.55)	0.675
Infection	12.5%	7.2%	1.84	(1.45, 2.33)	< .001	7.1%	6.2%	1.17	(0.81, 1.70)	0.400
*With Adjuvant Chemotherapy*										
Overall Complication	39.1%	22.6%	2.20	(1.83, 2.64)	< .001	22.8%	20.8%	1.13	(0.85, 1.51)	0.413
Anastomotic leak	11.1%	7.0%	1.65	(1.24, 2.21)	0.001	7.3%	8.0%	0.90	(0.58, 1.40)	0.644
Ileus	15.0%	8.2%	1.98	(1.53, 2.57)	< .001	8.3%	10.0%	0.81	(0.55, 1.21)	0.309
Bleeding	13.4%	9.2%	1.52	(1.17, 1.98)	0.002	9.3%	10.5%	0.87	(0.59, 1.28)	0.474
Infection	16.0%	8.7%	2.00	(1.56, 2.58)	< .001	8.4%	7.8%	1.09	(0.70, 1.69)	0.706

IPTW, inverse probability of treatment weighting; MIS, minimally invasive surgery; LS, laparoscopic surgery; RS, robotic surgery; OR, odds ratio; CI, confidence interval

## Discussion

In this study, we analyzed a national claims dataset to compare short- and long-term health-care expenditures by surgical approach to colon cancer over a 1-year period. Not unexpectedly, this study showed that patients who received adjuvant chemotherapy, regardless of surgical platform, had higher expenditures at all time points compared to those who did not receive adjuvant chemotherapy. Open surgery utilized higher short- and long-term expenditures than LS or RS in both the AC- and AC+ groups. The total mean expenditure difference was $6677 higher for OS compared to MIS in the AC- patients and $36,048 for AC + group. When comparing LS and RS, there were no differences between the platforms for each of the AC- and AC+ groups at the index operation. However, the LS group had higher expenditures than RS in the 30-day post-surgery period in both the AC- and AC+ groups, and over the 1-year study period following the index operation, mean health-care expenditures were higher for LS than RS in the AC- group.

These findings further support the relative value of MIS in colorectal surgery. OS shows higher expenditures up to 1-year post-index surgery. This reflects both the expenditures at the time of the index operation (increase in complications, longer length of stay) as well as additional expenditures incurred as a direct result of laparotomy (pain, opiate use, hernia, longer time to return to optimal normal function)[3, 6, 7, 9].

As has been demonstrated by other investigators, the MIS group demonstrated a lower perioperative complication rate compared to OS [6, 10, 15]. Health-care expenditure at 30-day post-discharge is shown in Supplementary Table 4. The expenditure difference between open and MIS colectomy were from inpatient and outpatient expenditures in both AC- (mean difference: inpatient, $732; outpatient, $173) and AC+ patients (mean difference: inpatient, $1334; outpatient, $1105). Within MIS, the observed higher expenditures for the LS approach than for RS were due to higher outpatient appointment cost among AC- patients (mean difference: $236) and higher inpatient admission expenditure among AC+ patients (mean difference: $761).

The added value of RS over LS was an interesting finding. The 30-day expenditure saving of RS may be explained by differences in extraction site incision length and location, different trocar trauma mechanics, the variable use of intracorporeal vs extracorporeal techniques for anastomosis, or other differences between the platforms. Studies have shown improved outcomes for intracorporeal anastomosis in MIS right colectomy [16, 17]. The use of intracorporeal anastomosis allows for less organ manipulation, smaller incision length, and placement of incisions off midline. Intracorporeal anastomosis can be done with LS or RS, but the uptake is very low in LS and very high in RS. This has implications regarding extraction site (pain and hernia risk). Conversion-to-open surgery is costly and has also been shown to be considerably higher in LS compared to RS in this and other studies [18–22].

The long-term value of RS in AC- patients may be related to extraction site hernia rates which have often been shown to be higher for LS than RS [23]. We would expect these outcomes to manifest beyond the 30-day period. One explanation of why this difference did not carry over to the AC+ group may be that the smaller, but statistically significant differences in expenditures may have been washed out by the substantially higher expenditures associated with administration of chemotherapy. Patients receiving chemotherapy may also delay elective treatment of incisional hernias unless significantly symptomatic and until adjuvant therapy is completed.

This large claims database study assessing mean total health-care expenditures includes data elements in the current United States health-care system that would be difficult to collect by other methods. The database contains data from commercial insurance and may be more comprehensive and generalizable than a single-institution study. However, these results may not be generalizable to the Medicare patient population. There may be selection bias when choosing between laparoscopic and robotic approaches that are unable to be controlled for in a large database analysis that may impact conversion and other outcomes. “Conversion via inspection” codes were used to capture conversion-to-open cases in the first year of the study when ICD10 codes for conversion were not available (Supplementary Table 2). This may not have captured all converted cases and may be a study limitation. This study stratified patients by their adjuvant chemotherapy status to focus on a homogeneous population with colon cancer to minimize bias when comparing between surgical approaches. However, the dataset is retrospective and does not include data elements that might have helped explain some of the differences between groups. It is also difficult to adjust for selection bias related to patient or disease factors, variable surgeon skill sets, access to resources, and identification of specific underlying sources of cost. We tried to mitigate these limitations by restricting the analysis to a uniform patient population with colon cancer undergoing elective surgery as the first treatment. We excluded emergency cases, known preoperative metastatic disease, and patients who had neoadjuvant chemotherapy. This study uniquely contributes to the understanding of real-world differences between the study groups and clarifies the total health-care expenditure implications choosing a surgical approach for patients with colon cancer.

When physicians and patients navigate the health-care expenditure implications of surgical approach decision-making, it is important for hospitals to collect and report both short- and long-term data that is all-encompassing. This database study allowed the inclusion of both inpatient and outpatient expenditures and stratification of pharmacy, intraoperative, physician, hospital, and patient expenses. Future studies that allow granular detail not provided in current claims databases are warranted.

## Conclusion

This study shows that there are short- and long-term cost advantages when choosing minimally invasive approaches to colon cancer. The value of improved outcomes at lower health-care expenditures supports the recommendation to select minimally invasive surgery whenever possible for elective colon cancer treatment. The robotic approach adds further savings over LS in the 30-day post-surgery period regardless of chemotherapy status; for patients who did not receive adjuvant chemotherapy, the health-care expenditure savings advantage extends over the 1-year study period following the index operation.

## Supplementary Information

Below is the link to the electronic supplementary material.

Supplementary file1 (DOCX 14 KB)

Supplementary file1 (DOCX 15 KB)

Supplementary file2 (DOCX 28 KB)

Supplementary file3 (DOCX 16 KB)
