# Supplementary material for: Health-care expenditures are less for minimally invasive than open colectomy for colon cancer: A US commercial claims database analysis
Source: Surg Endosc. 2023 May 16;37(8):6278–87. doi: 10.1007/s00464-023-10104-y (PMC10338385; doi:10.1007/s00464-023-10104-y)
Supplement: Supplementary file 1 — Supplementary file1 (DOCX 14 KB) [file 464_2023_10104_MOESM1_ESM.docx]

Supplementary Table 2: Conversion and Complication Codes

| **Measure** | **Grouping** | **Code type** | **Code** |
| --- | --- | --- | --- |
| Conversion | Conversion | ICD-9-CM | V64.41 |
|  |  | ICD-10-CM | Z53.31, Z53.39 |
|  | Conversion via Inspection | ICD-10-PCS | 0DJD4ZZ |
| Anastomotic Leak | N/A | ICD-9-CM | 567.22, 569.81, 997.4, 997.49 |
|  |  | ICD-10-CM | K63.2, K65.1, K91.81, K91.89 |
| Ileus | N/A | ICD-9-CM | 560.1 |
|  |  | ICD-10-CM | K56.0, K56.7, K91.30, K91.31 |
| Bleeding | N/A | ICD-9-CM | 285.1, 459.0, 578.9, 578.1, 998.11, 998.12 |
|  |  | ICD-10-CM | D62, R58, K91.61, K91.62, K91.840, K91.841, K91.870, K91.871, K92.1, K92.2 |
| Infections | N/A | ICD-9-CM | 998.51, 998.59, 567.22, 567.38, 567.39, 569.5, 567, 567.1, 567.21, 567.29, 567.89, 567.9 |
|  |  | ICD-10-CM | K63.0, K65.0, K65.1, K65.8, K65.9, K67, K68.11, K68.19, K68.9, T81.4XXX |

ICD-9-PCS/ICD-10-PCS, International Classification of Diseases, 9th and 10th Procedure Classification System; CPT, Current Procedural Terminology; ICD-9-CM/ICD-10-CM, International Classification of Diseases, 9th and 10th Clinical Modification.

^1^ Acute Post-hemorrhagic Anemia and Hemorrhage/Hematoma

^2^ Surgical Site Infections and Septicemia
