# Supplementary material for: Health-care expenditures are less for minimally invasive than open colectomy for colon cancer: A US commercial claims database analysis
Source: Surg Endosc. 2023 May 16;37(8):6278–87. doi: 10.1007/s00464-023-10104-y (PMC10338385; doi:10.1007/s00464-023-10104-y)
Supplement: Supplementary file 2 — Supplementary file1 (DOCX 15 KB) [file 464_2023_10104_MOESM2_ESM.docx]

Supplementary Table 3: Baseline demographic characteristics after inverse probability of treatment weighting (IPTW) adjustment.

|  | Patients without Adjuvant Chemotherapy | | | | | | |  | | Patients with Adjuvant Chemotherapy | | | | | | |
| --- | --- | --- | --- | --- | --- | --- | --- | --- | --- | --- | --- | --- | --- | --- | --- | --- |
|  | Open  (N=885) | MIS  (N=3531) | SMD |  | LS  (N=2966) | RS  (N=560) | SMD | |  | Open  (N=752) | MIS  (N=1894) | SMD |  | LS  (N=1554) | RS  (N=337) | SMD |
|  | N (%) | N (%) |  |  | N (%) | N (%) |  | |  | N (%) | N (%) |  |  | N (%) | N (%) |  |
| Age, years |  |  | 0.01 |  |  |  | 0.02 | |  |  |  | 0.01 |  |  |  | 0.01 |
| 18-44 | 76.7 ( 8.7) | 295.7 ( 8.4) |  |  | 245.8 ( 8.3) | 43.1 ( 7.7) |  | |  | 107.3 (14.3) | 276.0 (14.6) |  |  | 226.1 (14.5) | 49.9 (14.8) |  |
| 45-54 | 316.4 (35.8) | 1275.9 (36.1) |  |  | 1131.0 (38.1) | 216.0 (38.6) |  | |  | 278.0 (37.0) | 708.0 (37.4) |  |  | 601.5 (38.7) | 130.5 (38.8) |  |
| 55-64 | 491.6 (55.6) | 1958.9 (55.5) |  |  | 1589.3 (53.6) | 300.4 (53.7) |  | |  | 366.6 (48.8) | 910.3 (48.1) |  |  | 726.5 (46.7) | 156.0 (46.4) |  |
| Gender, Male | 443.3 (50.1) | 1748.5 (49.5) | 0.01 |  | 1470.6 (49.6) | 280.2 (50.1) | 0.01 | |  | 395.2 (52.6) | 986.6 (52.1) | 0.01 |  | 825.7 (53.1) | 177.4 (52.7) | 0.01 |
| Region |  |  | 0.02 |  |  |  | 0.08 | |  |  |  | 0.01 |  |  |  | 0.13 |
| North Central | 199.4 (22.5) | 784.0 (22.2) |  |  | 642.5 (21.7) | 117.0 (20.9) |  | |  | 165.0 (21.9) | 420.6 (22.2) |  |  | 332.9 (21.4) | 72.7 (21.6) |  |
| Northeast | 135.6 (15.3) | 557.9 (15.8) |  |  | 485.7 (16.4) | 103.8 (18.6) |  | |  | 111.0 (14.8) | 278.1 (14.7) |  |  | 244.3 (15.7) | 59.4 (17.7) |  |
| South | 423.7 (47.9) | 1685.9 (47.8) |  |  | 1396.9 (47.1) | 265.5 (47.4) |  | |  | 371.7 (49.4) | 934.0 (49.3) |  |  | 750.9 (48.3) | 161.9 (48.1) |  |
| West | 120.8 (13.7) | 484.2 (13.7) |  |  | 424.2 (14.3) | 71.6 (12.8) |  | |  | 98.9 (13.2) | 248.1 (13.1) |  |  | 216.0 (13.9) | 42.4 (12.6) |  |
| Unknown | 5.2 ( 0.6) | 18.5 ( 0.5) |  |  | 16.8 ( 0.6) | 1.7 ( 0.3) |  | |  | 5.3 ( 0.7) | 13.7 ( 0.7) |  |  | 9.8 ( 0.6) | 0.0 ( 0.0) |  |
| Metropolitan |  |  | 0.01 |  |  |  | 0.02 | |  |  |  | 0.01 |  |  |  | 0.03 |
| Yes | 714.3 (80.7) | 2856.6 (80.9) |  |  | 2452.5 (82.7) | 465.2 (83.1) |  | |  | 604.4 (80.4) | 1515.4 (80.0) |  |  | 1271.5 (81.8) | 276.8 (82.3) |  |
| No | 129.9 (14.7) | 511.2 (14.5) |  |  | 368.4 (12.4) | 69.3 (12.4) |  | |  | 118.0 (15.7) | 303.3 (16.0) |  |  | 220.1 (14.2) | 48.1 (14.3) |  |
| Unknown | 40.6 ( 4.6) | 162.7 ( 4.6) |  |  | 145.2 ( 4.9) | 25.1 ( 4.5) |  | |  | 29.6 ( 3.9) | 75.7 ( 4.0) |  |  | 62.4 ( 4.0) | 11.6 ( 3.4) |  |
| Insurance Plan |  |  | 0.02 |  |  |  | 0.03 | |  |  |  | 0.01 |  |  |  | 0.05 |
| PPO | 479.8 (54.2) | 1932.3 (54.7) |  |  | 1630.1 (55.0) | 304.9 (54.5) |  | |  | 408.2 (54.3) | 1033.1 (54.5) |  |  | 866.6 (55.8) | 183.0 (54.4) |  |
| Capitated plan | 111.1 (12.6) | 441.5 (12.5) |  |  | 358.2 (12.1) | 70.6 (12.6) |  | |  | 95.8 (12.7) | 238.5 (12.6) |  |  | 192.3 (12.4) | 39.9 (11.8) |  |
| Comprehensive | 46.3 ( 5.2) | 168.5 ( 4.8) |  |  | 144.6 ( 4.9) | 30.6 ( 5.5) |  | |  | 33.4 ( 4.4) | 83.0 ( 4.4) |  |  | 57.8 ( 3.7) | 12.9 ( 3.8) |  |
| Non-cap POS | 70.0 ( 7.9) | 286.2 ( 8.1) |  |  | 237.4 ( 8.0) | 43.3 ( 7.7) |  | |  | 59.9 ( 8.0) | 155.5 ( 8.2) |  |  | 125.9 ( 8.1) | 29.8 ( 8.9) |  |
| Others | 162.5 (18.4) | 640.4 (18.1) |  |  | 540.4 (18.2) | 100.0 (17.9) |  | |  | 142.5 (18.9) | 354.4 (18.7) |  |  | 289.0 (18.6) | 64.7 (19.2) |  |
| Unknown | 15.1 ( 1.7) | 61.5 ( 1.7) |  |  | 55.4 ( 1.9) | 10.1 ( 1.8) |  | |  | 12.1 ( 1.6) | 29.9 ( 1.6) |  |  | 22.4 ( 1.4) | 6.2 ( 1.9) |  |
| Surgical site |  |  | 0.02 |  |  |  | 0.01 | |  |  |  | 0.00 |  |  |  | 0.03 |
| Right | 534.9 (60.5) | 2162.4 (61.3) |  |  | 1855.0 (62.5) | 346.6 (62.0) |  | |  | 377.8 (50.2) | 947.5 (50.0) |  |  | 793.7 (51.1) | 167.4 (49.8) |  |
| Left |  |  |  |  |  |  |  | |  |  |  |  |  |  |  |  |
| IBD | 20.5 ( 2.3) | 79.5 ( 2.3) | 0.00 |  | 61.6 ( 2.1) | 9.3 ( 1.7) | 0.03 | |  | 10.7 ( 1.4) | 28.1 ( 1.5) | 0.01 |  | 19.8 ( 1.3) | 4.8 ( 1.4) | 0.01 |
| Polyps | 377.2 (42.6) | 1515.9 (42.9) | 0.01 |  | 1294.4 (43.6) | 247.1 (44.2) | 0.01 | |  | 172.1 (22.9) | 436.8 (23.1) | 0.00 |  | 356.9 (23.0) | 70.0 (20.8) | 0.05 |
| Diverticular | 68.6 ( 7.8) | 263.7 ( 7.5) | 0.01 |  | 213.3 ( 7.2) | 40.8 ( 7.3) | 0.00 | |  | 43.0 ( 5.7) | 112.9 ( 6.0) | 0.01 |  | 91.5 ( 5.9) | 23.6 ( 7.0) | 0.05 |
| DRG |  |  | 0.20 |  |  |  | 0.05 | |  |  |  | 0.21 |  |  |  | 0.07 |
| 329 | 89.3 (10.1) | 176.2 ( 5.0) |  |  | 139.0 ( 4.7) | 31.1 ( 5.6) |  | |  | 81.1 (10.8) | 125.5 ( 6.6) |  |  | 104.1 ( 6.7) | 20.4 ( 6.0) |  |
| 330 | 621.6 (70.3) | 2541.1 (72.0) |  |  | 2134.2 (72.0) | 391.4 (70.0) |  | |  | 624.3 (83.0) | 1570.5 (82.9) |  |  | 1281.5 (82.5) | 285.8 (84.9) |  |
| 331 | 173.7 (19.6) | 813.1 (23.0) |  |  | 692.9 (23.4) | 137.0 (24.5) |  | |  | 46.5 ( 6.2) | 198.3 (10.5) |  |  | 168.4 (10.8) | 30.3 ( 9.0) |  |
| Charlson Comorbidity |  |  | 0.01 |  |  |  | 0.02 | |  |  |  | 0.01 |  |  |  | 0.05 |
| 0 | 496.2 (56.1) | 1984.7 (56.2) |  |  | 1674.3 (56.4) | 321.9 (57.5) |  | |  | 418.9 (55.7) | 1062.8 (56.1) |  |  | 860.4 (55.4) | 190.8 (56.7) |  |
| 1 | 246.8 (27.9) | 995.0 (28.2) |  |  | 856.4 (28.9) | 157.5 (28.2) |  | |  | 226.1 (30.1) | 565.3 (29.8) |  |  | 482.1 (31.0) | 96.5 (28.7) |  |
| >=2 | 141.7 (16.0) | 550.9 (15.6) |  |  | 435.4 (14.7) | 80.1 (14.3) |  | |  | 107.0 (14.2) | 266.2 (14.1) |  |  | 211.4 (13.6) | 49.1 (14.6) |  |
| Overweight/Obesity | 180.7 (20.4) | 696.6 (19.7) | 0.02 |  | 579.8 (19.5) | 108.2 (19.3) | 0.01 | |  | 128.2 (17.0) | 320.7 (16.9) | 0.00 |  | 260.4 (16.8) | 57.9 (17.2) | 0.01 |
| Year |  |  | 0.03 |  |  |  | 0.02 | |  |  |  | 0.02 |  |  |  | 0.04 |
| 2013 | 120.2 (13.6) | 473.3 (13.4) |  |  | 376.5 (12.7) | 68.7 (12.3) |  | |  | 106.3 (14.1) | 267.7 (14.1) |  |  | 203.3 (13.1) | 41.8 (12.4) |  |
| 2014 | 167.5 (18.9) | 667.9 (18.9) |  |  | 558.8 (18.8) | 104.2 (18.6) |  | |  | 139.3 (18.5) | 352.1 (18.6) |  |  | 267.9 (17.2) | 54.4 (16.2) |  |
| 2015 | 168.7 (19.1) | 665.7 (18.9) |  |  | 542.1 (18.3) | 103.5 (18.5) |  | |  | 124.9 (16.6) | 311.5 (16.4) |  |  | 250.0 (16.1) | 56.2 (16.7) |  |
| 2016 | 127.4 (14.4) | 520.9 (14.8) |  |  | 431.9 (14.6) | 81.7 (14.6) |  | |  | 115.4 (15.3) | 289.9 (15.3) |  |  | 222.6 (14.3) | 50.3 (14.9) |  |
| 2017 | 109.8 (12.4) | 464.6 (13.2) |  |  | 401.5 (13.5) | 75.3 (13.5) |  | |  | 93.2 (12.4) | 244.8 (12.9) |  |  | 211.8 (13.6) | 44.7 (13.3) |  |
| 2018 | 94.0 (10.6) | 371.9 (10.5) |  |  | 327.7 (11.0) | 63.2 (11.3) |  | |  | 91.3 (12.1) | 223.7 (11.8) |  |  | 203.9 (13.1) | 45.1 (13.4) |  |
| 2019 | 97.1 (11.0) | 366.0 (10.4) |  |  | 327.7 (11.0) | 62.9 (11.2) |  | |  | 81.4 (10.8) | 204.8 (10.8) |  |  | 194.4 (12.5) | 44.0 (13.1) |  |
| Baseline total payment | $15033 ± $18023 | $14825 ± $20072 | 0.01 |  | $14590 ± $19366 | $14287 ± $16014 | 0.02 | |  | $15892 ± $20172 | $15719 ± $18192 | 0.01 |  | $15329 ± $17736 | $15349 ± $14825 | 0.00 |

IPTW, inverse probability of treatment weighting; SMD, standard mean difference; MIS, minimally invasive surgery; LS, laparoscopic surgery; RS, robotic surgery; PPO, preferred provider organization; Non-cap POS, Non-capitated Point-of-Service; DRG, Diagnosis Related Group; DRG 329/330/331, major small and large bowel procedures. DRG codes were listed but not included in IPTW model.
