# Supplementary material for: Health-care expenditures are less for minimally invasive than open colectomy for colon cancer: A US commercial claims database analysis
Source: Surg Endosc. 2023 May 16;37(8):6278–87. doi: 10.1007/s00464-023-10104-y (PMC10338385; doi:10.1007/s00464-023-10104-y)
Supplement: Supplementary file 3 — Supplementary file2 (DOCX 28 KB) [file 464_2023_10104_MOESM3_ESM.docx]

Supplementary Table 4. Breakdown of IPTW-adjusted differences in 30-day post-discharge health care expenditures

|  | Open | | MIS | | Difference  (Open - MIS) | |  | LS | | RS | | Difference  (LS - RS) | |
| --- | --- | --- | --- | --- | --- | --- | --- | --- | --- | --- | --- | --- | --- |
|  | Mean | 95%CI | Mean | 95%CI | Mean | P-Value |  | Mean | 95%CI | Mean | 95%CI | Mean | P-Value |
| Without Adjuvant Chemotherapy | |  |  |  |  |  |  |  |  |  |  |  |  |
| Inpatient expenditure | 2042 | (1686, 2473) | 1310 | (1190, 1442) | 732 | 0.001 |  | 1366 | (1230, 1516) | 1096 | (863, 1394) | 269 | 0.078 |
| Outpatient expenditure | 1357 | (1205, 1530) | 1184 | (1115, 1257) | 173 | 0.055 |  | 1214 | (1137, 1297) | 978 | (841, 1137) | 236 | 0.006 |
| Pharmacy expenditure | 263 | (231, 300) | 256 | (240, 274) | 7 | 0.721 |  | 256 | (239, 275) | 204 | (173, 240) | 53 | 0.007 |
| With Adjuvant Chemotherapy | |  |  |  |  |  |  |  |  |  |  |  |  |
| Inpatient expenditure | 2940 | (2384, 3626) | 1606 | (1407, 1833) | 1334 | <.001 |  | 1694 | (1466, 1958) | 933 | (684, 1273) | 761 | <.001 |
| Outpatient expenditure | 8010 | (7304, 8784) | 6905 | (6515, 7318) | 1105 | 0.010 |  | 6916 | (6487, 7373) | 6184 | (5389, 7095) | 732 | 0.134 |
| Pharmacy expenditure | 476 | (414, 546) | 515 | (472, 561) | -39 | 0.332 |  | 491 | (446, 541) | 584 | (475, 718) | -92 | 0.163 |

IPTW, inverse probability of treatment weighting; MIS, minimally invasive surgery; LS, laparoscopic surgery; RS, robotic surgery; CI, confidence interval.
