# Supplementary material for: Health-care expenditures are less for minimally invasive than open colectomy for colon cancer: A US commercial claims database analysis
Source: Surg Endosc. 2023 May 16;37(8):6278–87. doi: 10.1007/s00464-023-10104-y (PMC10338385; doi:10.1007/s00464-023-10104-y)
Supplement: Supplementary file 4 — Supplementary file3 (DOCX 16 KB) [file 464_2023_10104_MOESM4_ESM.docx]

Supplementary Table 1: Procedure codes used for eligibility

| **Measure** | **Grouping** | **Code type** | **Code** |
| --- | --- | --- | --- |
| Left colectomy | Laparoscopic | ICD-9- PCS | 17.35, 17.36 |
|  |  | ICD-10-PCS | 0DTM4ZZ, 0DTG4ZZ, 0DTN4ZZ |
|  | Open | ICD-9- PCS | 45.75, 45.76 |
|  |  | ICD-10-PCS | 0DTM0ZZ, 0DTG0ZZ, 0DTN0ZZ |
| Right colectomy | Laparoscopic | ICD-9- PCS | 17.32, 17.33 |
|  |  | ICD-10-PCS | 0DTF4ZZ, 0DTH4ZZ, 0DTK4ZZ |
|  | Open | ICD-9- PCS | 45.72, 45.73 |
|  |  | ICD-10-PCS | 0DTF0ZZ, 0DTH0ZZ,0DTK0ZZ |
| Robotic surgery | N/A | CPT | S2900 |
|  |  | ICD-9- PCS | 17.4x |
|  |  | ICD-10-PCS | 8E0**CZ |
| Colon Cancer | N/A | ICD-9- PCS | 153.X, 154.0, 209.1X, 209.30, 230.3, 230.4 |
|  |  | ICD-10-PCS | C18.X, C19, C7A02X, C7A.8, D01.X |

ICD-9-PCS/ICD-10-PCS, International Classification of Diseases, 9th and 10th Procedure Classification System; CPT, Current Procedural Terminology; ICD-9-CM/ICD-10-CM, International Classification of Diseases, 9th and 10th Clinical Modification.
